# Genomic and proteomic profiling I: Leiomyomas in African Americans and Caucasians

**DOI:** 10.1186/1477-7827-5-34

**Published:** 2007-08-23

**Authors:** Qun Pan, Xiaoping Luo, Nasser Chegini

**Affiliations:** 1 Department of Obstetrics and Gynecology, University of Florida, Gainesville, Florida 32610, USA

## Abstract

**Background:**

Clinical observations indicate that leiomyomas occur more frequently in African Americans compared to other ethnic groups with unknown etiology. To identify the molecular basis for the difference we compared leiomyomas form A. Americans with Caucasians using genomic and proteomic strategies.

**Methods:**

Microarray, realtime PCR, 2D-PAGE, mass spectrometry, Western blotting and immunohistochemistry.

**Results:**

Using Affymetrix U133A array and analysis based on P ranking (P < 0.01) 1470 genes were identified as differentially expressed in leiomyomas compared to myometrium regardless of ethnicity. Of these, 268 genes were either over-expressed (177 genes) or under-expressed (91 genes) based on P < 0.01 followed by 2-fold cutoff selection in leiomyomas of A. Americans as compared to Caucasians. Among them, the expression E2F1, RUNX3, EGR3, TBPIP, ECM2, ESM1, THBS1, GAS1, ADAM17, CST6, CST7, FBLN5, ICAM2, EDN1 and COL18 was validated using realtime PCR low-density arrays. 2D PAGE coupled with image analysis identified 332 protein spots of which the density/volume of 31 varied by greater than or equal to 1.5 fold in leiomyomas as compared to myometrium. The density/volume of 34 protein-spots varied by greater than or equal to 1.5 fold (26 increased and 8 decreased) in leiomyomas of A. Americans as compared to Caucasians. Tandem mass spectrometric analysis of 15 protein spots identified several proteins whose transcripts were also identified by microarray, including 14-3-3 beta and mimecan, whose expression was confirmed using western blotting and immunohistochemistry.

**Conclusion:**

These findings imply that the level rather than the ethnic-specific expression of a number of genes and proteins may account for the difference between leiomyomas and possibly myometrium, in A. Americans and Caucasians. Further study using larger sample size is required to confirm these findings.

## Background

It has been estimated that 70% of women have a life-long risk of developing leiomyomas, with symptomatic tumors accounting for a 1/3 of all the hysterectomies preformed annually in the United States alone. Such estimates are even higher among African Americans with leiomyomas that develop earlier, become larger and more symptomatic as compared to other ethnic groups [1-3]. Since leiomyomas develop during the reproductive years and regress with menopause, ovarian steroids are critical to their growth, however the molecular environment to explain these ethnic differences is unknown.

Various conventional and recent large-scale gene expression-profiling studies have provided valuable information regarding the molecular environment of leiomyomas [4-11]. Most of these studies were based on comparing leiomyomas with myometrium obtained from different ethnic groups without examining the influence of ethnicity on overall gene expression [9,10,12]. Using tissue microarray a recent immunohistochemistry study has reported the expression of a number of proteins in leiomyomas with some differences in their expression intensities in leiomyomas from African Americans as compared to other ethnic groups [13]. In addition, an increased risk of developing leiomyomas, including among African Americans, has been examined in a number of genetic studies, although in a majority of the cases the evidence of genomic instability is inconsistent [14-17].

Considering the above information and availability of limited data differentiating leiomyomas microenvironment in African Americans in the present study we combined genomic and proteomic strategies comparing the molecular environment of paired leiomyoma and myometrium from African Americans with Caucasians. Furthermore, from microarray data sets we selected 15 differentially expressed genes from different functional categories rather than levels of their expression and validated their expression using realtime PCR in a microfluidic card format. Additionally, 15 protein spots identified by proteomic were isolated and subjected to tandem mass spectrometry (MS/MS) for identification of their content and verified two of these proteins by Western blotting and immunohistochemistry.

## Methods

Portions of leiomyoma and matched myometrium were collected from six women, ranging from 29 to 38 years old who were scheduled to undergo hysterectomy for indications related to symptomatic leiomyomas. Three of the patients were Caucasians and three African Americans. These women were not taking any medication including hormonal therapy for the pervious 3 months prior to surgery and based on their last menstrual period and endometrial histology they were from early-mid secretory phase of the menstrual cycle. The myometrium used in this study was collected from regions distal from leiomyomas. All the leiomyomas were subserosal/intramural and 3 to 4 cm in diameter. These tissues were collected at the University of Florida affiliated Shands Hospital with prior approval from the Institutional Review Board without requiring to obtain informed consent. The tissues were snapped frozen and stored in liquid nitrogen until used.

### Microarray and gene expression profiling

Small portions of the above tissues were used to isolate total RNA using Trizol (Invitrogen, Carlsbad, CA). RNA quality and yield was analyzed with Agilent 2100 Bioanalyzer (Agilent Technologies, Foster City, CA). RNA amplification was carried out as previously described [7], and following second-strand cDNA synthesis 5 μg of purified cDNA was reverse transcribed using Enzo BioArray high yield RNA transcript labeling kit (Affymetrix, Santa Clara, CA). After purifying the product, 20 μg of cRNA (0.5 μg/μl) was fragmented. The fragment then mixed with 300 μl of hybridization mixture and 200 μl of the mixture was hybridized to human U133A Affymetrix GeneChip which consists of 22,277 oligonucleotide probe sets representing 18,400 transcripts and variants, including 14,500 known genes. The chips were processed after meeting recommended criteria for use of the expression arrays as previously described [7] and according to manufactures protocol.

### Microarray data analysis

The chips were scanned using Affymetrix Genepix 5000A scanner and the resulting images were assessed using Genepix software with a manual supervision to detect any inaccuracies. The net hybridization values were determined and subjected to global normalization and transformed expression values were subjected to Affymetrix Analysis Suite V 5.0 to remove any probe sets that were flagged as absent on all arrays using default settings. The expression value of the remaining probe sets was then subjected to unsupervised and supervised learning, discrimination analysis, and cross validation as previously described [7]. After variation filtering, the coefficient of variation was calculated for each probe set across all chips, ranked and the expression values of the selected genes were then subjected to statistical analysis in "R" programming as previously described [7]. The gene expression values having a statistical significance of p ≤ 0.01 (ANOVA, Turkey test) were selected and subjected to 2-fold cutoff. Hierarchical clustering and TreeView analysis was carried out and the selected genes were subjected to functional annotation and visualization using Database for Annotation, Visualization, and Integrated Discovery [18] software. The integrated GoCharts assigns genes to specific ontology functional categories based on selected classifications.

### Low-density microfluidic card and realtime PCR

Total RNA isolated from the same paired leiomyomas and myometrium from African Americans and Caucasians used for microarray analysis was also subjected to realtime PCR using microfluidic cards TaqMan Low Density Arrays (LDAs) assessing the level of expression of 23 selected genes, and the house-keeping gene, G3PDH. These genes were selected based on their functional categories rather than the level of their expression from the list of differentially expressed genes identified in the present and our previous [6,7,19] microarray studies. The products of these 23 genes have been implicated in several biological processes involved in pathogenesis of leiomyoma and other fibrotic disorders. Pre-designed TaqMan probe and primer sets for these genes were selected from the on-line catalogue and were factory-loaded into customized 384-well LDA plates (Applied Biosystem, Foster City, CA). The LDA format was arranged on-line with each array containing two replicates for each 23-target gene and GAPDH. Since these LDAs were used to determine the expression of these genes in several different tissues for establishing the least variable housekeeping gene as standard prior to these experiments we utilized a factory-loaded LDA (384-well plate) specific for 12 housekeeping genes. The results indicated that GAPDH expression displayed the least variation among the reference genes in these tissues as well as the tissues used in the accompanied manuscript [20]. Based on these results we selected GAPDH as a housekeeping gene during the development of the LDA and for normalization of the expression of other target genes on LDA. The exact locations and the sequences of the oligonucleotides used in all assays can be downloaded from the Applied Biosystems website [21] by selecting the Assays IDs. All genes span exonintron boundaries and cover the major transcript forms.

The LDAs were analyzed using the 7900HT system with a TaqMan LDA Upgrade (Applied Biosystems). In brief, 5 μl of single-stranded cDNA at final concentration of 2 μg starting RNA was combined with 45 μl water and 50 μl TaqMan Universal PCR Master Mix. The mixture was injected into selected sample ports and the cards were centrifuged twice for 1 min at 1200 rpm and sealed to prevent well-to-well contamination. Thermal cycling conditions were as follows: 50°C for 2 min, 95°C for 10 min, 97°C for 30s and 60°C for 1 min for 40 cycles. In all the experiments total RNA isolated from each sample were assayed in duplicate and one sample was used in all cards to assess inter- and intra-assay variability. Since each LDA card has a unique barcode the Sequence Detection System plate documents stores the information on the plate type, detector, sample/target gene configurations, thermal cycling conditions, data collection, and raw fluorescence data at each cycle. The data obtained from each card was analyzed using the comparative method and following normalization of expression values to GAPDH expression using relative quantity (RQ) and Sequence Detection Software 2.2.1 for automated data analysis according to the manufacturer's guidelines [21]. For calculating the RQ of selected mRNA in leiomyomas compared with myometrium, or tissues from African Americans compared to Caucasians, myometrial RNA used in each card was designated as calibrator. To analyze the RQ amounts of selected genes as an effect of ethnicity, Caucasian myometrium was used as calibrators. The Ct value >35 was selected as cutoff for the absence of gene expression. The expression of 15 of these genes is reported here.

### Proteomic: protein isolation and CyDye labeling

Total protein was isolated from 100 to 300 mg of paired frozen leiomyoma and myometrium from African Americans and Caucasians. The tissues were grinded in liquid nitrogen until fine powder, 5 ml of Trizol reagent and 5 to 15 μl of protease inhibitor cocktail (Sigma-Aldrich, St. Louis, MO) was added and centrifuged at 10,000 × g for 20 min at 4°C. Following phase extraction with chloroform the mixture was centrifuged at 5000 RPM at 4°C for 20 min, the inter- and bottom phases were extracted with 100% ethanol and clarified by centrifugation. The protein was precipitated with isopropanol overnight at -20°C and the pellets were washed twice with ice cold 80% ethanol, air dried and dissolved in 300 μl of CyDye labeling buffer (8 M urea, 2 M thiourea, 4% CHAPS, 20 mM Tris pH 8.5) and centrifuged at 40,000 × g for 30 min. The supernatants were transferred to a clean tube and adjusted to pH of 8.5 and protein concentration was determined using EZQ^® ^(Invitrogen). Fifty μg of myometrial and leiomyomas protein mixtures was labeled with 400 pM of Cy3 and Cy5 respectively, for 30 min on ice in dark and the reaction was stopped and quenched with 1 μl of 10 mM Lysin.

### Two-dimensional electrophoresis

For the first dimension, the Cy3- and Cy5-labeled proteins were combined with 450 μg each of unlabeled protein and 100 mM of DTT ampholyte buffer pH 3 to 11, to 0.5% and adjusted the final volume to 400 μl. An 18 cm of pH 3 to 11 immobilized pH gradient (IPG) strip was passively rehydrated with above sample solution overnight in the dark. Isoelectrofocusing (IEF) was carried in IPGphor (Amersham, GE Healthcare Bio-Sciences Corp. Piscataway, NJ) and the proteins co-migrated together was focused at 8000 V at 20°C for 60 kV hr. For the second dimension SDS-PAGE, an 8 to 16% precast Tris Glycine gel (BioRad, CA) was used. Prior to mounting, the IPG strips were reduced and equilibrated in 15 ml of 50 mM Tris-HCl pH 6.8, 6 M Urea, 30% (v/v) glycerol 2% (w/v) SDS 100 mM DTT for 15 min, then was alkylated with 15 ml of 50 mM Tris-HCl pH 6.8 6 M Urea 30% (v/v) glycerol 2% SDS 2.5% idoacetamide for 15 min. 2nd dimension was carried in the dark at 20°C for 4 to 5 hrs at 24 mA/gel. MW markers (New England Biolabs, Beverly, MA) were added to the 2D-PAGE.

### Image acquisition and spot quantification

The gels were scanned with Typhoon 9400 Variable Mode Imager (Amersham) and following image acquisition the protein spots were identified, their volumes were determined and analyzed using computer-assisted gel analysis and Progenesis PG220 2D software (Nonlinear Dynamics, Durham, NC). The analysis allows automatic detection and quantification of protein spots as well as resizing, alignment, and matching between different 2D gel images. A database of all protein spots obtained from digital images was created, and integrated intensity of each spot was calculated based on spot area and optical density and expressed as percent volume, namely, spot volume/volumes of all spots resolved in the gel. The comparative program was used to geometrically correct spatial differences and allow comparison between the images. The information from comparison of the image databases was then used to establish an average gel. Each protein spot volume was normalized against the total protein spots volume present in the gel and the fold difference of each protein was calculated for the paired myometrium and leiomyoma sample. The list of protein spots with assigned identification number was prepared and arranged according to the fold difference and the values were statistically analyzed using ANOVA, Turkey test.

### Protein identification by mass spectrometric analysis

The gels were Coomassie blue stained and the spots of interest were identified and manually excised with a disposable gel puncher and stored in 25% methanol until tyrptic digestion according to a protocol used by Proteomics Core Facility of the Inter-disciplinary Cores for Biomedical Research (ICBR) at the University of Florida. Peptides derived from tryptic digestion were analyzed by reversed-phase HPLC-tandem mass spectrometry on a hybrid quadrupole time-of-flight instrument (QSTAR XL, Applied Biosystems) equipped with a nano-electrospray source. Solvent delivery at 200 nl/min was provided by an integrated capillary HPLC system (Ultimate, LC Packings, Sunnyvale, CA) in which a 30 min gradient from 3% to 50% acetonitrile in 0.1% acetic acid was employed. Each information dependent acquisition (IDA) cycle consisted of a survey scan from m/z 400 to 1500 and three MS/MS scans obtained by collision-induced dissociation of ions that demonstrated the largest signal intensity at a given chromatographic time point. Survey and MS/MS scans were accumulated for 1 and 2 sec, respectively.

Tandem mass spectrometric data were searched against the IPI human protein database using the Mascot search algorithm. Carbamidomethylation of cysteine was included as a fixed modification whereas oxidation of methionine, deamidation of asparagine and glutamine, and pyro-glu formation from N-terminal glutamine or glutamic acid were included as variable modifications. Probability-based MOWSE scores that exceeded the value corresponding to p < 0.05 were considered for protein identification. After inputting the pI and MW of proteins Swiss-Prot Database was searched by TagIdent software (Swiss Institute of Bioinformatics, Basel, Switzerland) for matching proteins.

### Western blot analysis

Briefly, small portions of paired leiomyoma and myometrial tissues were homogenized in homogenizing buffer containing protease inhibitor cocktail [22]. The laysets were centrifuged, supernatants protein content was determined and an equal amount of proteins was subjected to SDS-PAGE, transfer by electroblotting and the blots were exposed to polyclonal antibodies generated against 14-3-3β and mimecan (Santa Cruz Biotechnology, Santa Cruz, CA), respectively and monoclonal antibody to β-actin (Sigma) to normalize for protein loading. The blots were exposed to corresponding HRP-conjugated IgG and visualized using enhanced chemiluminesence reagents (Amersham). The band intensity was determined using Kodak gel analysis software (Eastman Kodak, Rochester, New York).

### Immunohistochemistry

For immunohistochemical localization of 14-3-3β and mimecan, fixed and paraffin embedded leiomyomas and myometrial tissue sections 3 to 5 μm thick were prepared. Following standard procedures, the sections were incubated with polyclonal antibodies at 5 μg of IgG/ml prepared in phosphate buffered saline, pH 7.4 [22]. The sections were than exposed to biotinylated second antibodies and avidin horseradish peroxidase (Vector Laboratories, Burlingame, CA) and chromogenic reaction was developed using 3, 3'diaminobenzidine and counter sections were counter staining with hemotoxalin as indicated. Tissue sections incubated with normal IgG instead of the primary antibodies, or deletion of the primary antibodies during immunostaining served as controls.

## Results

### Gene expression profiling of leiomyomas from African Americans and Caucasians

**Figure 1:**
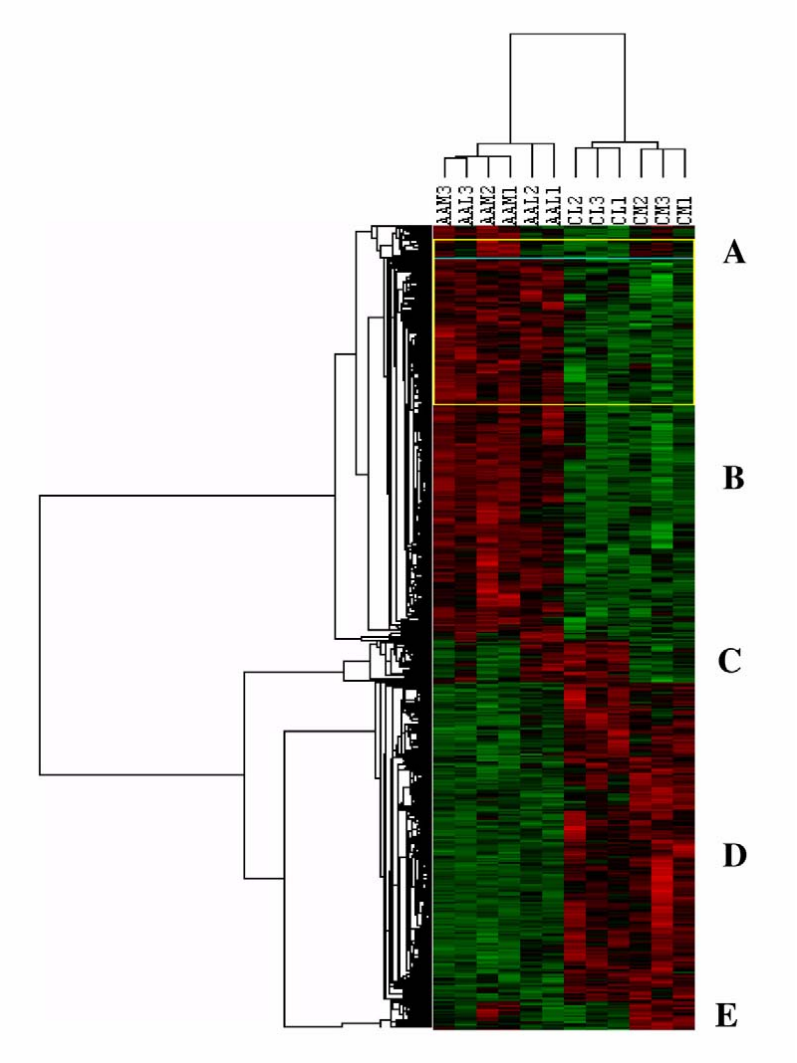
Hierarchical cluster analysis of differentially expressed genes in matched leiomyoma and myometrium from African Americans (AAL1, AAL2, AAL3, AAM1, AAM2, AAM3) and Caucasians (CL1, CL2, CL3, CM1, CM2, CM3) selected at P ≤ 0.01 followed by 2-fold cutoff change. Each column represents data from a single cohort with shades of red and green indicating up- or down-regulated genes according to the color scheme shown below. Genes represented by rows were clustered according to their similarities in pattern of expression in each tissue. The dendrogram at the top of the image displays similarity in gene expression among the cohorts, and relatedness of the arrays is denoted by distance to the node linking the arrays. The clustering divided the genes into five clusters designated as A to E.

Gene expression values obtained from microarray analysis of paired leiomyoma and myometrium from African Americans and Caucasians were subjected to unsupervised and supervised learning. Based on statistical analysis with p ranking of P ≤ 0.01 we identified 1470 transcripts, or 8% of the genes on the array, as differentially expressed among these tissues regardless of ethnicity. Further analysis based on 2-fold cutoff resulted in identification of 268 genes with 177 genes over-expressed and 91 genes under-expressed in leiomyomas of African Americans as compared to Caucasians (Table 1; for the complete list of genes see Table 4, Additional file 1). Hierarchical clustering separated these genes into several distinctive clusters (Fig. 1) and functional pathway analysis indicated that majority of their products serve as transcriptional, translational and signal transduction mediators, cell cycle regulation, ECM turnover, cell-cell communication and metabolic activities etc.

**Table 1:** Differentially expressed genes in leiomyomas of African Americans as compared to Caucasians

Gene Bank	Gene Symbol	Probability	Fold Change	Biological Function
P43115	PTGER3	0.007	3.05	signal transduction
NM_006549	CAMKK2	0.0004	2.66	signal transduction
Q92844	TANK	0.00002	2.57	signal transduction
NM_000267	NF1	0.0004	2.57	signal transduction
L35253	MAPK14	0.0011	2.51	signal transduction
Q16825	PTPN21	0.004	2.51	signal transduction
P04156	PRNP	0.00003	2.33	signal transduction
P27986	PIK3R1	0.004	2.19	signal transduction
NM_002835	PTPN12	0.0001	2.18	signal transduction
P04901	GNB1	0.0002	2.02	signal transduction
NM_005456	MAPK8IP1	0.006	0.50	signal transduction
P17612	PRKACA	0.003	0.50	signal transduction
NM_003331	TYK2	0.001	0.50	signal transduction
P17010	ZFX	0.0004	3.73	transcription factor
O00712	NFIB	0.00002	2.56	transcription factor
NM_002040	GABPA	0.004	2.38	transcription
U18671	STAT2	0.001	2.20	transcription
AF040963	MXD4	0.0002	0.50	transcription
Q9Y6K9	IKBKG	0.004	0.50	transcription
L41066	NFATc4	0.001	0.48	transcription
P26196	DDX6	0.008	0.48	RNA processing
O95997	PTTG1	0.009	0.37	oncogenes/tumor suppressors
NM_012141	DDX26	0.0009	0.31	tumor suppressor
P10159	EIF5A	0.0004	2.63	translation
NM_001969	EIF5	0.0001	2.15	translation
NM_000141	FGFR2	0.002	12.66	growth factor receptor
NM_005761	PLXNC1	0.002	3.48	cell receptor
P36897	TGFBR1	0.006	4.81	growth factor receptor
D50683	TGFBR2	0.006	3.54	growth factor receptor
O60462	NRP2	0.002	2.43	cell receptors
O94816	FZD7	0.004	2.04	cell receptors
M26062	IL2RB	0.002	0.46	cell receptors
NM_004883	NRG2	0.007	0.46	cell signaling
M59040	CD44	0.002	2.70	cell surface antigens
P07585	DCN	0.007	2.00	cell surface antigens
P30408	TM4SF1	0.004	2.00	cell surface antigens
M_002456	MUC1	0.008	0.48	cell surface antigens
Q9NVA2	FLJ10849	0.002	3.55	cell cycle
P54826	GAS1	0.001	2.12	cell cycle
Q14004	CDC2L5	0.0001	2.05	cell cycle
NM_003644	GAS7	0.005	0.50	cell cycle
P29466	CASP1	0.008	2.28	apoptosis
NM_016315	GULP1	0.0007	2.04	apoptosis
NM_006595	API5	0.0004	2.02	apoptosis
Q01082 S	PTBN1	0.00005	4.52	cytoskeleton/motility
NM_005909	MAP1B	0.002	3.01	cytoskeleton/motility
P35749	MYH11	0.0005	2.88	cytoskeleton/motility
Q13642	FHL1	0.002	2.59	cytoskeleton/motility
Q9NYL9	TMOD3	0.0002	2.39	cytoskeleton/motility
P35222	CTNNB1	0.005	2.04	cell adhesion receptors
NM_006158	NEFL	0.0006	0.10	cytoskeleton/motility
NM_004995	MMP14	0.003	3.96	protein turnover
NM_013381	TRHDE	0.008	2.33	protein turnover
	LTBP1	0.003	0.50	protein kinase activity
NM_000849	GSTM3	0.0002	7.99	metabolism
NM_000850	GSTM4	0.005	2.36	metabolism
P06733	ENO1	0.005	2.07	metabolism
P08294	SOD3	0.0005	0.44	metabolism
NM_004177	STX3A	0.0007	0.53	trafficking/targeting proteins
Q9NZ08	ARTS-1	0.008	2.20	stress response proteins
AF112465	OGN	0.003	3.05	membrane channels
NM_000725	CACNB3	0.001	0.54	membrane channels
L22548	COL18A1	0.002	0.51	membrane channels
	PTGIS	0.009	2.58	lipid biosynthesis

Partial list of genes from several functional categories differentially expressed in leiomyomas of African Americans as compared to Caucasians as illustrated in figure 1 and selected based on p ≤ 0.001 and 2-fold cutoff change (F. Change) as described in materials and methods. The complete list is provided as in Additional file 1.

### Gene expression using TaqMan LDA

**Figure 2:**
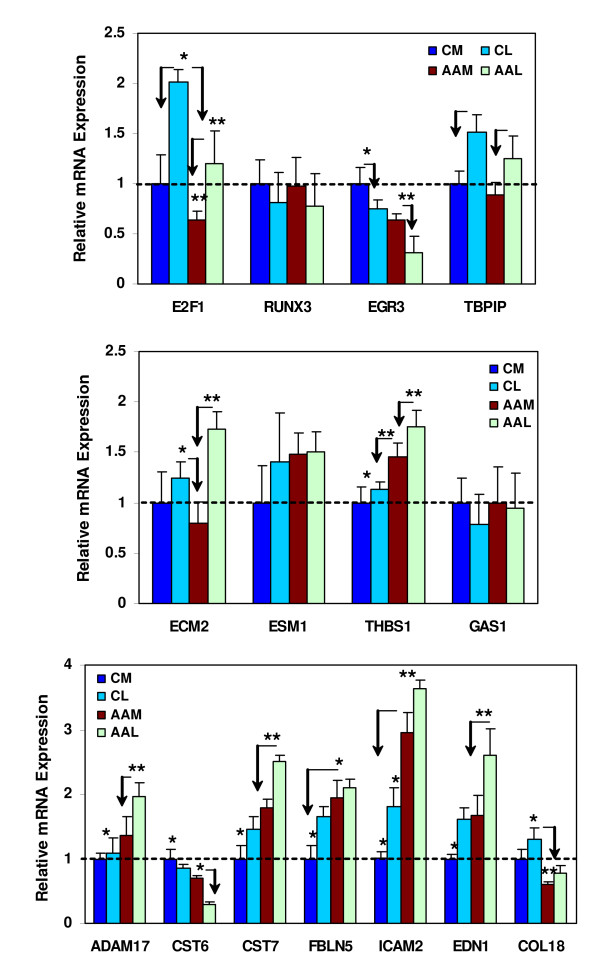
Bar graphs show the relative level of expression of 15 of the differentially expressed genes in paired myometrium (M) and leiomyomas (L) from Caucasians (C) and African Americans (AA) determined by realtime PCR low density array. Values on the y-axis represent an arbitrary unit derived from the mean expression of each gene independently with the mean value of myometrium from Caucasian set at 1 for each gene. The asterisks * are statistically different from ** comparing paired myometrium and leiomyomas from African Americans and Caucasians with arrows pointing out the difference between the expression of these genes in leiomyoma and myometrium in each group. A probability level of P < 0.05 was considered significant.

We used customized 384-well LDA cards to determine the expression of 15 genes in leiomyomas and myometrium, with each card containing two replicates of the target genes and GAPDH as a housekeeping gene (Fig. 2). Since these cards were also used to assess the expression of these genes in tissues reported in the accompanied manuscript [20] we first established the least variable housekeeping gene to use as a standard among these tissues. Using factory-loaded LDA cards representing 12 housekeeping genes GAPDH expression displayed the least variation among these tissues (data not shown). Based on these results we selected GAPDH as a housekeeping gene and along with 23 target genes for preparation of customized LDA for our study. The selection of the 23 genes was based on the result of the present and our previous microarray studies [6,7,19]. Figure 2 show the level of expression of E2F1, RUNX3, EGR3, TBPIP, ECM2, ESM1, THBS1, GAS1, ADAM17, CST6, CST7, FBLN5, ICAM2, EDN1 and COL18, representing transcription factors, cell cycle and apoptosis regulators, matrix remodeling and cell adhesion signaling, respectively in matched leiomyoma and myometrium from African Americans and Caucasians. The level of expression of these genes varied significantly in leiomyoma and myometrium as well as tissues from African Americans as compared to Caucasians (Fig. 2; P < 0.05), some displaying a similar pattern of expression observed with microarray.

### Proteomic analysis of leiomyomas and myometrium

**Figure 3:**
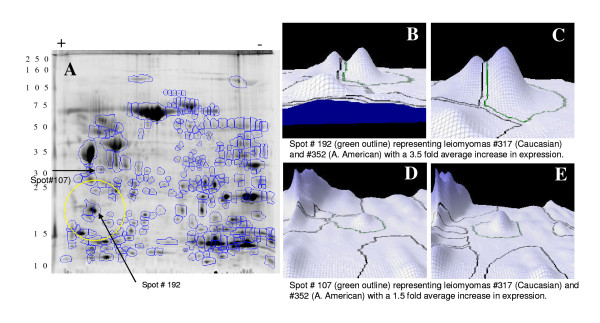
Two-dimensional gel electrophoresis of total protein isolated from leiomyoma and myometrium. Protein spots with differentiate expression are encircled and identified by a number. The spots were identified using MALDI-TOF and peptide matching. Figures B to E show the three-dimensional images with differential expression of two protein spots (#107 and 192) in leiomyomas of African American (C and E) and Caucasians (B and D)

A representative of total protein isolated from a paired myometrium and leiomyoma, labeled with Cy3 or Cy5 respectively, and co-separated in 2D PAGE is shown in Figure 3A. Using image analysis 332 protein spots were identified and their density/volume determined with two spots (#107 and #192) later extracted for identification shown in graphic presentation (Fig. 3B). The average density of each protein spot in leiomyomas and myometrium was normalized against their density in one of the myometrium from a Caucasian patient. Of the 332 protein spots identified 28 and 31 spots were unique to leiomyomas and myometrium, respectively with the density/volume of 31 spots varied by at least 1.5 fold (12 were overexpressed and 19 underexpressed) in leiomyomas as compared to myometrium regardless of ethnicity. 109 protein spots identified in leiomyomas the average density/volume of 34 spots differed by at least 1.5 fold, of which 26 spots were overexpressed and 8 were underexpressed in African American as compared to Caucasians (Table 2). Mass spectrometric analysis of 15 selected spots resulted in identification of 137 proteins. A database search against Swiss-Prot resulted in identification of several proteins and the results are summarized in Table 3, with MS and MS/MS scores showing the reliability of identification. Among the content of these protein spots included several keratins, Annexin A1 and V, transgelin (SM22), galgizzarin (S100A11) and EF-hand domin-containing protein 2 (S100A12), vimentin, retinoic acid binding protein II, cofilin, several isoforms of 14-3-3 and mimecan. Transcripts corresponding to these proteins were also identified among differentially expressed genes in leiomyomas and myometrium of both ethnic groups (Table 3).

**Table 2:** Profile of protein spots detected in leiomyomas from Caucasians and African Americans

**Tissue**	**Increased Intensity**	**Decreased Intensity**	**Not Changed**	**Not-Detected**
Fibroid-315C	31 (1.7–8.90 fold)	289 (1.5–48.80 fold)	-----	
Fibroid-316C	42 (1.5–13.2 fold)	171 (1.5–46.00 fold)	61	55
Fibroid-317C	129 (1.5–14.8 fold)	135 (1.5–128.7 fold)	3	64
Fibroid-344AA	180 (1.5–41.6 fold)	62 (1.5–37.70 fold)	7	81
Fibroid-353AA	81 (1.5–35.3 fold)	100 (1.5–86.80 fold)	33	118
Fibroid-357AA	80 (1.5–14.1 fold)	65 (1.5–38.20 fold)	22	165

The number of protein spots detected in leiomyomas from Caucasians (315C, 316C and 317C) and African Americans (334AA, 353AA and 357AA) as compared to protein spots detected in myometrium of 315C used for normalization. Total number of spots detected in myometrium #315 was 332. The column shows the number of spots whose intensity increased, decreased, not changed (± 1.5 fold), or not detected as compared to myometrium #315C. The range of difference in protein spot intensity is shown as fold change as compared to their intensity in myometrium 315C.

**Table 3:** List of protein spots and their contents identified by MS/MS in leiomyomas and myometrium

**Protein Spots**	**Description**	**Accession Number**	**Theoretical MW**	**Theoretical pI**	**Score**	**Match**	Transcripts *
76	Annexin A1	P04083	38.6	6.64	361	10	+
	Aflatoxin B1 Aldehyde reductase 2	O43488	39.6	6.70	359	11	+
	Splice Isoform 1	P31942	36.9	6.37	327	9	+
	Heterogeneous nuclear ribonucleoprotein H3						
	PDZ and LIM domain protein 1	O00151	35.9	6.55	257	5	+
	PTD012 protein	Q6FI88	35.1	6.23	217	7	+
	LIM and SH3 domain protein 1	Q14847	29.7	6.61	114	3	+
93	CGI-150 protein	Q9Y3E8	55.0	8.89	495	13	+
	Inorganic pyrophosphatase	Q9H2U2	38.0	7.07	106	3	+
107	Annexin V	P08758	35.8	4.94	619	23	+
	Mimecan	P20774	33.9	5.46	329	6	+
	Microtubule-associated protein RP/EB1	Q15691	29.9	5.02	180	4	+
	EF-hand domain-containing protein 2	Q96C19	26.7	5.15	155	3	+
	Tubulin-specific chaperone B	Q99426	27.3	5.06	132	3	+
126	14-3-3ε	Q04917	28.1	4.76	339	8	+
	14-3-3τ	P27348	27.8	4.68	317	7	+
	14-3-3γ	P61981	28.2	4.80	231	8	+
	14-3-3β/α	P31946	28.0	4.76	170	6	+
	Annexin V	P08758	35.8	4.94	162	4	+
	Proteasome α5	P28066	26.4	4.74	157	4	
	Vimentin	P08670	53.5	5.06	124	3	+
	Acidic leucine-rich nuclear phosphoprotein 32A	P39687	28.6	3.99	92	2	+
183	α crystallin B chain	P02511	20.2	6.76	489	28	+
187	Transgelin (SM22)	Q5U0D2	22.6	8.87	632	28	+
192	Myosin regulatory light chain 2, smooth muscle isoforms	P24844	19.7	4.8	333	15	+
	Myosin regulatory light chain 2, nonsarcomeric	P19105	19.7	4.67	224	9	+
204	Splice Isoform CFL2b of	Q9Y281	18.7	7.66	263	10	+
	Cofilin, muscle isoforms						+
	Cofilin, non-muscle isoforms	P23528	18.4	8.26	172	6	+
	Transgelin (SM22)	Q01995	22.5	8.88	96	3	+
206	Transgelin (SM22)	Q01995	22.5	8.88	329	14	+
	Destrin alpha				309	17	+
209	Actin-related protein 2/3	O15511	16.2	5.47	245	14	+
	complex subunit 5						
243	Retinoic acid-binding protein II, cellular	P29373	15.6	5.43	208	19	+
	14 kDa phosphohistidine phosphatase	Q9NRX4	13.8	5.65	105	3	+
303	Calgizzarin (S100A11)	P31949	11.7	6.56	164	10	+
	Cytochrome c oxidase polypeptide Vib				64	4	+
324	Ubiquitin	P62988	8.56	6.56	203	17	+

The identified proteins and their corresponding MASCOT scores obtained by 2D-PAGE separation combined with MALDI-TOFMS PMF and nano-HPLC-ESI-MS/MS peptide sequencing. Spot numbers corresponds to data in Fig. 3 with changes in their expression reported in Table 2. The corresponding transcripts (*) of the proteins identified by microarray in leiomyomas and myometrium at p ≤ 0.05 and 2-fold cutoff are shown as + in the right hand side column. The Accession numbers, theoretical MW and pI are according to and calculated from Swiss-Prot.

### Western blot analysis and immunohistochemical localization

**Figure 4:**
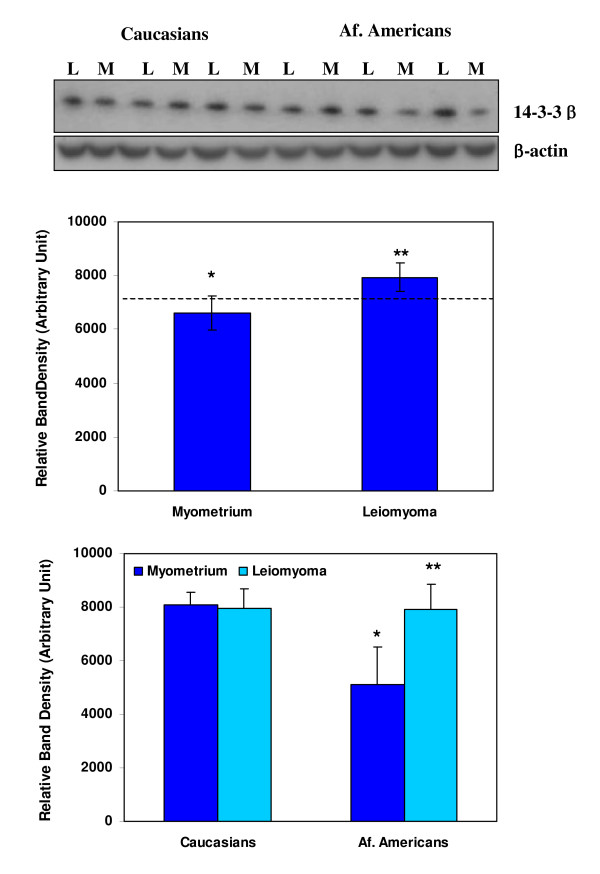
Western blot analysis of 14-3-3β (30 Kd) and β-actin (control) in paired leiomyoma (L) and myometrium (M) from African Americans (Af. American) and Caucasians. The bar graph shows the relative expression (band density) of 14-3-3β in leiomyomas and myometrium in ethnic-dependent and independent manners. The asterisks ** are statistically different from * (P < 0.05).

Western blot analysis indicated that leiomyoma and myometrium from both ethnic groups express 14-3-3β with a considerable variation among these tissues (Figs. 4). We were unable to detect mimecan immunoreactive protein in these tissue extracts by western blotting possibly because of the nature of the antibody for this application.

**Figure 5:**
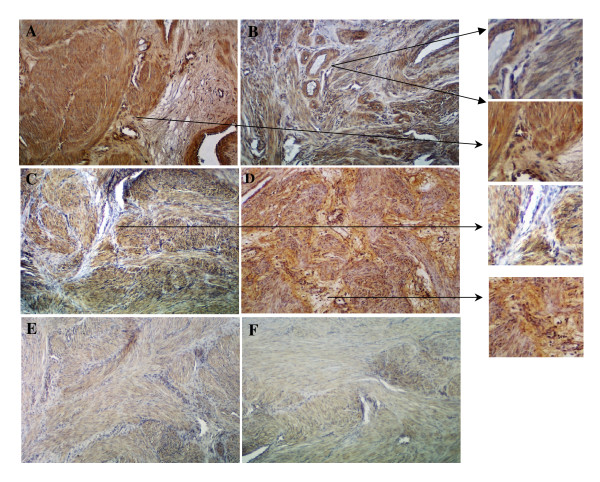
Immunohistochemical localization of 14-3-3β (A and B) and mimecan (C and D) in myometrium (A and C) and leiomyomas (B and D) associated with leiomyoma and myometrial smooth muscle cells and cellular components of connective tissue and vasculature with inserts showing a higher magnification portion of these tissues. Incubation of tissue sections with non-immune rabbit (E) and goat (F) IgGs instead of primary antibodies during immunostaining, served as controls, reduced the staining intensity. Mag: X150; inserts = X265

Immunohistochemically, 14-3-3β (Fig. 5A and 5B) and mimecan (Fig. 5C and 5D) were localized in leiomyoma and myometrial smooth muscle cells, connective tissue fibroblasts and vascular cells, with a considerable variability in their intensity among these cells within the same tissue (Figs. 5). Incubation of the tissue sections with normal rabbit and/or goat serum instead of the primary antibodies resulted in considerable reduction in staining intensity (Fig. 5E and 5F; the insert images show a higher magnification portion of these figures indicated by the arrows).

## Discussion

Using genomic and proteomic strategies the present study provided further insight into the molecular environments of leiomyoma and myometrium in Caucasians and African Americans. At genomic level 1470 genes or about 10% of the transcripts on the array were identified as differentially expressed in leiomyomas as compared to myometrium regardless of ethnicity. Of these genes 268 were identified as either over-expressed (177 genes) or under-expressed (91 genes) in leiomyomas of African Americans as compared to Caucasians. Hierarchical cluster analysis separated these genes into several clusters reflecting their tissue as well as ethnic-dependent and -independent association. However, the profile of some of the genes in these clusters displayed an area of relatedness between myometrium and leiomyomas within each ethnic group. This suggests that some of the differences in leiomyoma's gene expression might be attributed to differences in myometrial gene expression between the ethnic groups, as well as the differences in leiomyomas vs. myometrium. Functional analysis indicated that the majority of these genes serve as transcriptional, translational and signal transduction mediators, cell cycle regulation, ECM turnover, cell-cell communication and transport/metabolic activities.

At proteomic level we identified several proteins displaying both ethnic-dependent and -independent profiles, with 34, or 10% of total protein spots identified, displaying altered intensity in leiomyomas of African Americans as compared to Caucasians. Tandem mass spectrometry analysis of 15 selected protein spots revealed an array of proteins as part of their content (Table 2) of which the transcripts of some of them, or their related proteins, were among the genes identified in myometrium and leiomyomas. Although the genomic and proteomic results of our study in part confirmed the previous reports involving leiomyomas and myometrium [8-10,12,13,19,23,24], previous microarray analysis did not consider the influence of ethnicity as part of their comparative assessment. We recognize that low sample size is a limitation of our study, however validating the expression of a large number of transcripts simultaneously in the same samples by quantitative realtime PCR (low density array) and identification and confirmation of two proteins, mimecan (osteoglycin) and 14-3-3β in paired leiomyoma and myometrium compensated for this limitation.

Of the genes functionally relevant to processes that contribute to characteristic and pathogenesis of leiomyomas was the identification of several components of TGF-β system and their elevated expression in leiomyomas of African Americans as compared to Caucasians. Based on this and our previous studies we consider over-expression of TGF-β and TGF-β receptors in leiomyomas of African Americans result in further amplification of their signaling, targeted transcription factors and downstream genes expression, which are already altered in leiomyomas as compared to myometrium [7,19,22,25-28]. In contrast, leiomyomas of African Americans expressed lower levels of LTBP-1, a member of fibrillin super-family that associates and stores TGF-β into the ECM. LTBPs consist of LTBP-1 to LTBP-4 [29] with LTBP-1 and -2 expression reported in leiomyomas [30,31]. Commencing our study a recent report provided evidence for the expression of LTBP-1 and FBN-1 in leiomyomas of three different sizes with higher levels of expression in the medium-sized (3–5 cm) tumors compared with myometrium in the proliferative phase, while FBN-1 mRNA expression was size-independent [31]. Although the results suggested that ECM turnover in leiomyomas might be size-dependent, the larger tumors undergo significant alteration in their tissue structure, specifically at the center that must be taken into account during such comparative analysis. Our results that LTBP-1 is expressed at lower levels in leiomyomas of African Americans as compared to Caucasians suggest that TGF-β upon secretion and activation becomes readily available for binding rather than being stored into the ECM, a mechanism that regulates local availability of many growth factors and cytokines, including TGF-β.

Plasmin, integrins, thrombospondin-1 (TSP-1), mannose 6-phosphate, and decorin are known to activate TGF-β [32-34] and their differential expression suggests the presence of regulatory mechanism that control TGF-β action in leiomyoma where it regulates the expression of several genes involved in tissue fibrosis [19,25-28,35]. Additionally, we identified TCF8/ZB-1, a member of zinc finger transcription factors that include Smad interacting protein 1 (SIP1/ZEB-2), as differentially expressed gene in leiomyomas of African Americans. TCF8 through interaction with Smad and recruitment co-activator (p300) and co-repressor (CtBP), is involved in ER and TGF-β receptors signaling [35-37]. Leiomyomas express these and several other transcription factors that interact with TCF8 including Runx, CITED, EGR1, EGR3, E2F1, Nurr77, c-Myc, Max, and Mad, whose expression is validated in this and our previous study [7,19]. These transcription factors regulate the expression of group of genes involved in cell cycle regulation, apoptosis, angiogenesis and inflammation mediated by the action of TGF-β receptor and ER activation [38-43]. Because leiomyomas grow more rapidly in African Americans as compared to other ethnic groups, altered expression of these genes regulated by TGF-β and ovarian steroids could influence the outcome of this process.

A balance between cell growth and apoptosis is critical in progression of tissue fibrosis and the expression of several genes functionally related to these categories, including Bcl-XL, Bad, Bax, p27Kip1, p57Kip2, Gas1, Gas7, CST6, CST7, caspases, etc., were identified in leiomyomas and myometrium. Differential expression of some of these genes in leiomyomas of African Americans most likely contributes toward their rapid growth as compared to other ethnic group. Local apoptotic and non-apoptotic cell-death also results in regulation of multiple genes involved in inflammation, angiogenesis, fibrogenesis and tissue turn over. Among these genes involved in these processes are collagens, versican, fibromodulin, syndecan 4, TSP-1, tenascin-C, osteonectin/SPARC and ECM2, endothelial cell specific molecule-1 (ESM-1), ICAM2, EDN1, FZD7, β1 catenin, CST6, CST7 as well as several member of integrins, MMPs, TIMPs and ADAMs [44-49] that are expressed in leiomyomas [7,26,27] with altered expression in African Americans. The identification of 14-3-3β and mimecan as differentially expressed genes in leiomyomas of African Americans is also of interest. 14-3-3s are regulatory proteins that bind to a variety of cellular targets, including Raf kinase, cell cycle-dependent phosphatase Cdc25, pro-apoptotic protein Bad and many others proteins and are considered to regulate hypertrophic scar formation through regulation of MMPs expression [50-52]. The ability of 14-3-3 proteins to bind and regulate various oncogenic gene products as well as various tumor suppressor gene products points to a potential role in cancer As such 14-3-3 has been shown to inhibit TSC1/TSC2 complex functions and overexpression of either TSC1 or TSC2 in Hela cells has been reported to increase the expression of various 14-3-3 isoforms [53] suggesting that deregulated expression of 14-3-3 and TSC can be associated with leiomyoma pathogenesis. Mimecan is a member of small leucine-rich proteoglycans family, which includes lumican, fibromodulin, decorin and biglycan, and has been implicated in collagen fibrillogenesis, cellular growth, and migration [54-56]. Mimecan is secreted and proteolytically cleaved by a serine protease to form the 25–30 kDa form found in association with ECM of connective tissue and considered to play a structural role by maintaining the tensile strength and hydrated nature of the tissue [55,56]. Mimecan is expressed in mouse pituitary gland and in human anterior pituitary gland and Pit-1 is reported to activate the human mimecan promoter through Pit-1 response element sites [57]. As such mime can and 14-3-3 as well as other genes products in these categories may have a similar biological activity in leiomyoma pathogenesis, specifically in African Americans with more symptomatic tumors.

Because the ovarian steroids are critical to fibroid growth it is essential to assess their relationship with genomic and proteomic profiling presented here since the expression of some of these genes are the target of ovarian steroids regulatory functions. Since the tissues used in our study come from the early-mid secretary phase of the menstrual cycle it is possible to assume that both estrogen and progesterone-dependent and independent mechanism influence the expression of these genes. Additionally, we only identified the content of 15 of the protein spots isolated from the proteomic protein profiling as compared with a large number of genes identified by microarry. This limited our ability to assess any overlapping expression between genomic and proteomic results which determine whether different genes or proteins are regulated at different molecular level since some genes are regulated at transcription level, while others at protein level.

In conclusion, subjecting paired leiomyoma and myometrium from African Americans and Caucasians to genomic and proteomic analysis we identified a considerable similarity between their molecular environments with differences seen at the level rather than ethnic-specific expression of a number of genes. The area of relatedness among some of the gene clusters between myometrial and leiomyomas within each ethnic group suggests that some of the differences in leiomyoma's gene expression might be attributed to differences in myometrial gene expression between the ethnic groups, in addition to differences in leiomyomas vs. myometrium. Because many of the differentially expressed genes identified in these cohorts are know to regulate inflammatory response, angiogenesis, cell cycle, apoptosis and ECM matrix turnover, their products may account for rapid growth and associated symptoms in African Americans as compared to other ethnic groups.

## Competing interests

The author(s) declare that they have no competing interests.

## Authors' contributions

QP, XL and NC participated in all aspect of the experiments presented here with the final microarray gene chips and two-d gels were performed at Interdisciplinary Center for Biotechnology Research at the University of Florida. Gene expression analysis performed by XL, proteomic analysis and immunohistochemistry by NC, realtime PCR and Western blot by XL and QP. All the authors read and approved the final manuscript.

## Supplementary Material

Additional file 1 Table 4 - Differentially expressed genes in leiomyoma of African Americans and Caucasians. The complete list of differentially expressed genes identified in leiomyomas from African Americans and Caucasians as illustrated in Figure 1 and reported in part in Table 1. The genes were selected based on p ≤ 0.001 and 2-fold cutoff change (F. Change) as described in materials and methods. Click here for file
