# Supplementary material for: Genomic and proteomic profiling I: Leiomyomas in African Americans and Caucasians
Source: Reprod Biol Endocrinol. 2007 Aug 23;5:34. doi: 10.1186/1477-7827-5-34 (PMC2063502; doi:10.1186/1477-7827-5-34)
Supplement: Additional file 1 — Table 4 - Differentially expressed genes in leiomyoma of African Americans and Caucasians. The complete list of differentially expressed genes identified in leiomyomas from African Americans and Caucasians as illustrated in Figure 1 and reported in part in Table 1. The genes were selected based on p ≤ 0.001 and 2-fold cutoff change (F. Change) as described in materials and methods. [file 1477-7827-5-34-S1.doc]

**Table 4:** Differentially expressed genes in leiomyoma of African Americans and Caucasians.

| Gene Bank Symbol F. Change P value Function |
| --- |
| NM_005803 FLOT1 0.0008 4.7 signal transduction  NM_003930 SCAP2 0.0013 4.4 signal transduction  O14632 HIPK3 0.0002 3.2 signal transduction  P43115 PTGER3 0.0076 3.1 signal transduction  SOS1 0.0006 3.0 signal transduction  NM_006449 CDC42EP3 0.0064 2.7 signal transduction  NM_006549 CAMKK2 0.0004 2.7 signal transduction  P17964 RAP2B 0.0006 2.6 signal transduction  Q92844 TANK 0.0000 2.6 signal transduction  NM_000267 NF1 0.0004 2.6 signal transduction  L35253 MAPK14 0.0011 2.5 signal transduction  Q16825 PTPN21 0.0047 2.5 signal transduction  MAP4K5 0.0000 2.4 signal transduction  P04156 PRNP 0.0000 2.3 signal transduction  NM_006420 ARFGEF2 0.0026 2.3 signal transduction  P27986 PIK3R1 0.0045 2.2 signal transduction  NM_002835 PTPN12 0.0001 2.2 signal transduction  Q15382 RHEB 0.0004 2.2 signal transduction  Q9ULC3 RAB23 0.0000 2.2 signal transduction  P39687 ANP32A 0.0024 2.1 signal transduction  M37712 CDC2L2 0.0022 2.1 signal transduction  P26442 AMFR 0.0100 2.1 signal transduction  M33336 PRKAR1A 0.0001 2.1 signal transduction  CALM1 0.0005 2.1 signal transduction  NM_001387 DPYSL3 0.0077 2.0 signal transduction  P04901 GNB1 0.0002 2.0 signal transduction  Q9UPA1 G3BP2 0.0001 2.0 signal transduction  NM_004253 PLAA 0.0069 2.0 signal transduction  Q13614 MTMR2 0.0009 2.0 signal transduction  NM_006861 RAB35 0.0055 2.0 signal transduction  KIAA1053 protein SAMD4 0.0082 2.0 signal transduction  O95704 APBB3 0.0007 0.5 signal transduction  NM_005456 MAPK8IP1 0.0063 0.5 signal transduction  P17612 PRKACA 0.0038 0.5 signal transduction  Q9Y6J0 CABIN1 0.0042 0.5 signal transduction  FLJ39249 0.0004 0.5 signal transduction  NM_012395 PFTK1 0.0004 0.5 signal transduction  NM_003331 TYK2 0.0014 0.5 signal transduction  L13738 ACK1 0.0007 0.5 signal transduction  NM_005160 ADRBK2 0.0079 0.5 signal transduction  P20336 RAB3A 0.0052 0.4 signal transduction  NM_004723 ARHGEF2 0.0010 0.4 signal transduction  Q15486 SMA3 0.0022 0.3 signal transduction  P17010 ZFX 0.0000 3.7 transcription  TCF8 0.0000 3.3 transcription  CDC5L 0.0010 2.8 transcription  O43311 MBNL1 0.0000 2.6 transcription  O00712 NFIB 0.0000 2.6 transcription  NM_002040 GABPA 0.0046 2.4 transcription  NM_003670 BHLHB2 0.0045 2.3 transcription  U18671 STAT2 0.0010 2.2 transcription  NM_006625 FUSIP1 0.0000 2.2 transcription  CREB1 0.0001 2.2 transcription  Q9Y2S3 TAF9L 0.0000 2.1 transcription  AF040963 MXD4 0.0002 0.5 transcription  NM_012384 GMEB2 0.0082 0.5 transcription  Q9UEG5 CIC 0.0035 0.5 transcription  Q9Y6K9 IKBKG 0.0042 0.5 transcription  Q15737 SUPT6H 0.0019 0.5 transcription  L41066 NFATC4 0.0095 0.5 transcription  O95503 CBX6 0.0002 0.5 transcription  Q14656 CXorf12 0.0012 0.4 transcription  NM_006532 ELL 0.0019 0.4 transcription  NM_000937 POLR2A 0.0047 0.3 transcription  MBNL2 0.0000 4.4 RNA processing  Q13243 SFRS5 0.0003 4.4 RNA processing  NM_005885 TEB4 0.0003 3.3 RNA processing  P34096 RNASE4 0.0094 2.3 RNA processing  O43809 CPSF5 0.0003 2.1 RNA processing  HNRPH1 0.0082 2.1 RNA processing  Q15428 SF3A2 0.0006 0.5 RNA processing  NM_006711 RNPS1 0.0020 0.5 RNA processing  P26196 DDX6 0.0082 0.5 RNA processing  Q9Y388 CGI-79 0.0004 0.5 RNA processing  HIP14 0.0052 2.2 tumor suppressors  Q07157 TJP1 0.0029 2.1 tumor suppressors  O95997 PTTG1 0.0096 0.4 tumor suppressors  NM_012141 DDX26 0.0010 0.3 tumor suppressors  P36406 ARFD1 0.0064 3.0 translation  EIF4EBP2 0.0005 2.9 translation  P10159 EIF5A 0.0000 2.6 translation  P26438 ARF6 0.0000 2.6 translation  15E1.2 0.0010 2.4 translation  NM_005907 MAN1A1 0.0001 2.2 translation  P23411 RPL38 0.0083 2.2 translation  NM_001969 EIF5 0.0001 2.1 translation  P06396 GSN 0.0079 2.0 translation  NM_005494 DNAJB6 0.0000 2.0 translation  NM_006048 UBE4B 0.0037 2.0 translation  NM_004184 WARS 0.0008 0.5 translation  NM_000345 SNCA 0.0001 0.3 translation  NM_000141 FGFR2 0.0016 12.7 cell receptors  P36897 TGFBR1 0.0006 4.8 cell receptors  D50683 TGFBR2 0.0061 3.5 cell receptors  NM_005761 PLXNC1 0.0020 3.5 cell receptors  O60462 NRP2 0.0022 2.4 cell receptors  M59964 KITLG 0.0077 2.1 cell receptors  O94816 FZD7 0.0044 2.0 cell receptors  M26062 IL2RB 0.0022 0.5 cell receptors  Q13491 GPM6B 0.0059 3.1 cell surface antigens  M59040 CD44 0.0016 2.7 cell surface antigens  Q04900 CD164 0.0017 2.3 cell surface antigens  P07585 DCN 0.0065 2.0 cell surface antigens  P30408 TM4SF1 0.0040 2.0 cell surface antigens  NM_000733 CD3E 0.0080 0.5 cell surface antigens  NM_002456 MUC1 0.0080 0.5 cell surface antigens  M19154 TGFB2 0.0052 2.1 growth factor  NM_004883 NRG2 0.0069 0.5 growth factor  MT1E 0.0011 2.4 protein binding  O75083 WDR1 0.0033 2.4 protein binding  Q9NZ08 ARTS-1 0.0077 2.2 protein binding  PNAS-4 0.0018 2.2 protein target  NM_018846 SBBI26 0.0000 2.2 protein binding  Q9NUP9 LIN7C 0.0000 2.1 protein binding  DDX3X 0.0000 2.1 protein binding  Q9NVA2 FLJ10849 0.0023 3.6 cell cycle  NM_018310 BRF2 0.0012 2.8 cell proliferation  NM_022731 NUCKS 0.0020 2.5 cell cycle  O15172 PSPHL 0.0068 2.4 cell cycle  NM_016250 NDRG2 0.0015 2.3 cell cycle  P54826 GAS1 0.0012 2.1 cell cycle  Q14004 CDC2L5 0.0001 2.0 cell cycle  NM_005255 GAK 0.0001 0.5 cell cycle  NM_003644 GAS7 0.0046 0.5 cell cycle  O14569 101F6 0.0023 0.4 cell cycle  PTEN 0.0088 3.0 apoptosis  Q07820 MCL1 0.0006 2.9 apoptosis  NM_013436 NCKAP1 0.0036 2.6 apoptosis  P29466 CASP1 0.0081 2.3 apoptosis  BCLAF1 0.0001 2.1 apoptosis  NM_016315 GULP1 0.0000 2.0 apoptosis  NM_006595 API5 0.0000 2.0 apoptosis  BCL2L1 0.0083 0.5 apoptosis  Q01082 SPTBN1 0.0000 4.5 cytoskeleton/motility proteins  NM_005909 MAP1B 0.0016 3.0 cytoskeleton/motility proteins  P35749 MYH11 0.0005 2.9 cytoskeleton/motility proteins  Q13642 FHL1 0.0020 2.6 cytoskeleton/motility proteins  Q9NYL9 TMOD3 0.0002 2.4 cytoskeleton/motility proteins  PICALM 0.0001 2.3 cytoskeleton/motility proteins  NM_007124 UTRN 0.0012 2.1 cytoskeleton/motility proteins  HIP1R 0.0001 0.5 cytoskeleton/motility proteins  NM_006158 NEFL 0.0006 0.1 cytoskeleton/motility proteins  ITGA1 0.0004 2.7 cell adhesion  PLEKHC1 0.0003 2.3 cell adhesion  P35222 CTNNB1 0.0051 2.0 cell adhesion  NM_003156 STIM1 0.0058 0.5 cell adhesion  Q9Y368 PARVB 0.0002 0.3 cell adhesion  NM_001219 CALU 0.0060 2.0 extracellular transport  P55058 PLTP 0.0044 0.5 extracellular transport  P07988 SFTPB 0.0021 0.5 extracellular transport  NM_004995 MMP14 0.0033 4.0 protein turnover  NM_013381 TRHDE 0.0085 2.3 protein turnover  P06870 LAMP1 0.0009 2.0 protein turnover  NRP2 0.0015 2.1 protein kinase activity  LTBP1 0.0028 0.5 protein kinase activity  LRRK1 0.0015 0.3 protein kinase activity  DKFZP564M182 0.0015 0.5 protein catabolism  Q9H9C5 USP36 0.0033 0.5 protein catabolism  NM_022350 LRAP 0.0004 0.3 protein catabolism  NM_000849 GSTM3 0.0002 8.0 metabolism  PTGIS 0.0090 2.6 metabolism  NM_001918 DBT 0.0007 2.4 metabolism  NM_000850 GSTM4 0.0048 2.4 metabolism  NM_016286 DCXR 0.0044 2.1 metabolism  O15269 SPTLC1 0.0005 2.1 metabolism  P06733 ENO1 0.0045 2.1 metabolism  NM_001921 DCTD 0.0000 2.1 metabolism  NM_006759 UGP2 0.0056 2.1 metabolism  SCP2 0.0048 2.1 metabolism  P48449 LSS 0.0081 0.5 metabolism  P40967 SILV 0.0041 0.5 metabolism  Q05932 FPGS 0.0030 0.5 metabolism  NM_000203 IDUA 0.0003 0.5 metabolism  P05186 ALPL 0.0091 0.5 metabolism  PLA2G6 0.0004 0.4 metablism  P08294 SOD3 0.0005 0.4 metabolism  Q9UFW9 CLIC4 0.0020 3.4 trafficking proteins  P54920 SNAP23 0.0001 3.1 trafficking proteins  AF112465 OGN 0.0028 3.1 trafficking proteins  P14543 NID 0.0084 2.9 trafficking proteins  P08240 SRPR 0.0000 2.8 trafficking proteins  CYBRD1 0.0018 2.8 trafficking proteins  O15126 SCAMP1 0.0006 2.7 trafficking proteins  NM_006544 SEC10L1 0.0000 2.6 trafficking proteins  CALD1 0.0000 2.6 trafficking proteins  Q9Y587 AP4S1 0.0000 2.5 trafficking proteins  TRA1 0.0025 2.4 trafficking proteins  NM_003763 STX16 0.0002 2.4 trafficking proteins  NM_007047 BTN3A2 0.0025 2.4 trafficking proteins  Q15836 VAMP3 0.0000 2.4 trafficking proteins  NM_006364 SEC23A 0.0002 2.1 trafficking proteins  NM_006364 SEC23A 0.0002 2.1 trafficking proteins  NM_002268 KPNA4 0.0001 2.1 trafficking proteins  P53621 COPA 0.0002 2.0 trafficking proteins  NM_003304 TRPC1 0.0006 2.0 trafficking proteins  RNP24 0.0021 2.0 trafficking proteins  ATP6V0E 0.0005 2 trafficking proteins  NM_000725 CACNB3 0.0013 0.5 trafficking proteins  NM_004177 STX3A 0.0007 0.5 trafficking proteins  NM_003624 RANBP3 0.0000 0.5 trafficking proteins  O00499 BIN1 0.0030 0.5 trafficking proteins  L22548 COL18A1 0.0023 0.5 trafficking proteins  NM_003045 SLC7A1 0.0073 0.5 trafficking proteins  NM_003488 AKAP1 0.0087 0.4 trafficking proteins  NM_016287 HP1-BP74 0.0000 3.9 DNA packaging  L20298 CBFB 0.0098 2.2 DNA binding  P49454 CENPF 0.0084 0.4 DNA binding  GRINL1A 0.0045 2.0 maintenance of ER location  NM_019892 INPP5E 0.0059 0.5 phosphatase activity  KIAA0276 0.0002 2.1 nucleus  Q9BQQ3 GORASP1 0.0009 0.4 Golgi apparatus  Hyalpha NC00 0.0017 3.4 defense response  Q09160 HLA-A 0.0072 2.3 defense response  P02686 MBP 0.0013 2.1 defense response  MHC-1A 0.0068 2.1 defense response  M11886 HLA-C 0.0010 2.9 defense response  SSH-3 0.0009 6.6 not classified  Q9NXB3 CDKAL1 0.0002 5.8 not classified  PALMD 0.0052 3.8 not classified  NM_016613 DKFZp434L142 0.0003 3.6 not classified  NM_001862 COX5B 0.0005 3.5 not classified  Q9P007 HSPC157 0.0072 3.2 not classified  MED8 0.0016 3.2 not classified  Q9NS11 C14orf117 0.0000 2.7 not classified  C6orf62 0.0000 2.7 not classified  Q9NVV2 FLJ10490 0.0056 2.6 not classified  MT1K 0.0061 2.6 not classified  NEK9 0.0021 2.5 not classified  MGC15419 0.0047 2.5 not classified  LAP1B 0.0000 2.3 not classified  SP329 0.0000 2.2 not classified  O94968 KIAA0894 0.0099 2.2 not classified  NM_017548 H41 0.0001 2.1 not classified  SWAP70 0.0031 2.0 not classified  SCARB2 0.0001 2.0 not classified  Q9Y471 CMAH 0.0058 2.0 not classified  NM_030755 TXNDC 0.0004 2.0 not classified  O75071 KIAA0494 0.0003 2.0 not classified  NM_019018 FLJ11127 0.0041 2.0 not classified  CYB5-M 0.0000 2.0 not classified  MAP3K2 0.0003 2.0 not classified  FLJ20896 0.0018 0.5 not classified  TM6SF2 0.0011 0.5 not classified  NM_024094 MGC5528 0.0094 0.5 not classified  FLJ00133 0.0028 0.5 not classified  TPSB2 0.0053 0.5 not classified  O75781 PALM 0.0014 0.5 not classified  MGC29816 0.0000 0.5 not classified  EZI 0.0095 0.5 not classified  TST 0.0007 0.5 not classified  KIAA0365 SFRS14 0.0011 0.5 not classified  LOC254531 0.0008 0.5 not classified  TPSB1 0.0045 0.5 not classified  FLJ11871 0.0026 0.5 not classified  TBC1D3 0.0015 0.5 not classified  Q9P0T5 LOC51236 0.0027 0.5 not classified  NM_017670 OTUB1 0.0002 0.5 not classified  TPSB2 0.0012 0.4 not classified  NM_019005 FLJ20323 0.0006 0.4 not classified  Q9H008 LHPP 0.0004 0.4 not classified  KIAA0792 KIAA0792 0.0053 0.4 not classified  Q15004 KIAA0101 0.0014 0.4 not classified  NM_017814 FLJ20174 0.0029 0.4 not classified  FLJ22843 0.0089 0.4 not classified  Q9NXC7 PQLC2 0.0094 0.3 not classified  SMA3 0.0022 0.3 not classified |

The list of 266 differentially expressed genes in leiomyoma of African Americans as compare with Caucasians selected at P<0.001 and 2-fold cut-off, in part presented as Table 1. Partial list of genes from several functional categories differentially expressed in leiomyomas of African Americans as compared to Caucasians as illustrated in figure 1, selected based on p≤0.001 and 2-fold cutoff change (F. Change) as described in materials and methods.
